# Acute ischaemia and gap junction modulation modify propagation patterns across Purkinje-myocardial junctions

**DOI:** 10.3389/fphys.2025.1540400

**Published:** 2025-05-06

**Authors:** Richard J. Jabbour, Elham Behradfar, Michael Debney, Anders Nygren, Adam Hartley, Igor Efimov, Mélèze Hocini, Nicholas S. Peters, Fu Siong Ng, Edward J. Vigmond

**Affiliations:** ^1^ National Heart and Lung Institute, Imperial College London, London, United Kingdom; ^2^ Department of Biomedical Engineering, University of Calgary, Calgary, AB, Canada; ^3^ Department of Electrical and Software Engineering, University of Calgary, Calgary, AB, Canada; ^4^ McCormick School of Engineering, Northwestern University, Chicago, IL, United States; ^5^ Bordeaux University Hospital (CHU), Electrophysiology and Ablation Unit, Pessac, France; ^6^ IHU Liryc, Heart Rhythm Disease Institute, Fondation Bordeaux Université, Bordeaux, France; ^7^ University Bordeaux, Lab IMB, UMR 5251, Talence, France

**Keywords:** purkinje system, Purkinje-myocardial junction, ischaemia, computer modelling, cardiac electrophysiology

## Abstract

**Background:**

The Purkinje network is essential for normal electrical impulse propagation in the heart but has also been implicated in ventricular arrhythmias. Previous experimental work has suggested that not all Purkinje-myocardial junctions (PMJs) are active at rest due to source-sink mismatch at the PMJs.

**Objective:**

We hypothesized that pathological conditions that cause gap junction uncoupling (e.g., acute ischaemia), would increase the number of active PMJs, leading to more complex activation patterns.

**Methods:**

We investigated this using a whole-heart intact Purkinje system preparation that allowed direct high-resolution endocardial mapping to interrogate PMJ function. Twelve (7 control, five rotigaptide) Langendorff-perfused hearts from New Zealand white rabbits were subjected to an ischaemia-reperfusion protocol and optically mapped. Computational modelling was performed to determine the effects of gap junction coupling on PMJ function, and on the complexity of endocardial activation.

**Results:**

During ischaemia, the percentage of right ventricle area activated within the first 5 ms decreased from baseline 62% ± 7% to 52% ± 8% during early ischaemia (p = 0.04), consistent with slowing of conduction. This was followed by a paradoxical increase in late-ischaemia (60% ± 8%) due to extra regions of early activation. Gap junction enhancement with rotigaptide during ischaemia abolished the aforementioned pattern. Parallel computational experiments replicated experimental findings only when the number of functional PMJs was increased during ischaemia. With more active PMJs, there were more breakthrough sites with increased complexity of activation, as also measured in biological preparations.

**Conclusion:**

Normally-quiescent PMJs can become active in the context of gap junction uncoupling during acute ischaemia. Pharmacological gap junction modulation may alter propagation patterns across PMJs and may be used as a therapeutic strategy for Purkinje system associated arrhythmias.

## 1 Introduction

The Purkinje network consists of specialized conduction tissue that plays a key role in the rapid transmission of electrical impulses across the ventricles to allow for more synchronous contraction (Mendez et al., 1970; Robinson et al., 1987). However, the Purkinje system has also been implicated in the pathogenesis of several ventricular arrhythmias. For example, fascicular ventricular tachycardia (VT) and bundle-branch reentry both utilize the Purkinje network as part of their reentrant circuits (Haissaguerre et al., 2016). Furthermore, ventricular arrhythmias can also be triggered by Purkinje cells, for example, in idiopathic ventricular fibrillation (VF), and targeted ablation of Purkinje fibers is a feasible treatment for idiopathic VF (Nakagawa et al., 1993; Haïssaguerre et al., 2002; Haissaguerre et al., 2016).

The Purkinje network is connected to ventricular myocardium via Purkinje-myocardial junctions (PMJs) (Boyden, 2018). It has been postulated that many PMJs are normally in a non-functional state as there is significant source-sink mismatch at many PMJs, with the current from small Purkinje fibers being insufficient to anterogradely activate the larger bulk of ventricular myocardium (Joyner, 1982; Overholt et al., 1984). The susceptibility of PMJ transmission to changes in local environment has also been demonstrated experimentally (Tan et al., 1989). Other preclinical work has suggested the existence of a transitional cell type, coupled to both Purkinje and ventricular muscle cells via short thin strands and with distinct action potentials. These cells act as a high resistance barrier that could modulate anterograde impulse propagation ​(Tranum-Jensen et al., 1991).

**FIGURE 1:**
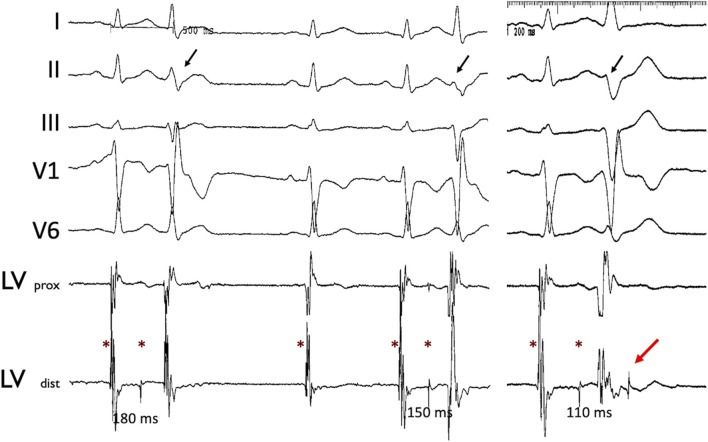
Concealed Purkinje discharge. Examples of ectopic beats arising from the LV Purkinje system, recorded during an invasive electrophysiological procedure in a patient. Left ventricle Purkinje beats with varying conduction times. Stars indicate the Purkinje potential. Note the varying Purkinje to ventricular muscle conduction times associated with the different QRS morphologies (black arrow) as well as non-conducted Purkinje beat (red arrow). Abbreviations: LV: left ventricle; prox: proximal; dist: distal.

Intracardiac recordings during catheter ablation in humans have confirmed that not all Purkinje potentials conduct into ventricular myocardium, supporting the source-sink mismatch hypothesis (Figure 1; central illustration). Partial gap junction uncoupling at the PMJs and of ventricular myocardium may improve the source-sink match at the PMJs to allow for propagation across previously-quiescent PMJs, as suggested indirectly in epicardial mapping studies in Cx43 knockout mice (Rohr et al., 1997; Morley et al., 2005).

We, therefore, hypothesized that pathological conditions that cause gap junction uncoupling, for example, acute ischaemia, may modify the source-sink relationship at PMJs sufficiently to allow for propagation of electrical impulses across previously-quiescent PMJs, leading to more complex activation patterns. We investigated this using a whole-heart intact Purkinje system preparation that allowed for direct high-resolution endocardial mapping to interrogate PMJ function. In parallel, we performed whole organ computational modelling to determine the effects of gap junction coupling on PMJ function, and on the complexity of endocardial activation.

## 2 Methods

### 2.1 Optical mapping

Experimental protocols were approved by the Washington University Institutional Animal Care and Use Committee, and by the Imperial College London Ethical Review Board (carried out under Project License PPL 70/7419), and were performed in accordance with standards set out in the United Kingdom Animals (Scientific Procedures) Act 1986.

Twelve Langendorff-perfused hearts from New Zealand white rabbits (∼4 months old, 2.0–2.5 kg) were studied in total. Briefly, after administration of anesthesia (sodium pentobarbital 30 mg/kg IV) and heparin, hearts were rapidly explanted and perfused on a Langendorff apparatus with oxygenated (95% O2-5% CO2) Tyrode’s solution (128.2 NaCl, 4.7 KCl, 1.19 NaH2PO4, 1.05 MgCl2, 1.3 CaCl2, 20.0 NaHCO3, and 11.1 glucose [all mmol/L], calibrated to pH = 7.35; 37°C; mean arterial pressure = 60 mmHg).

**FIGURE 2:**
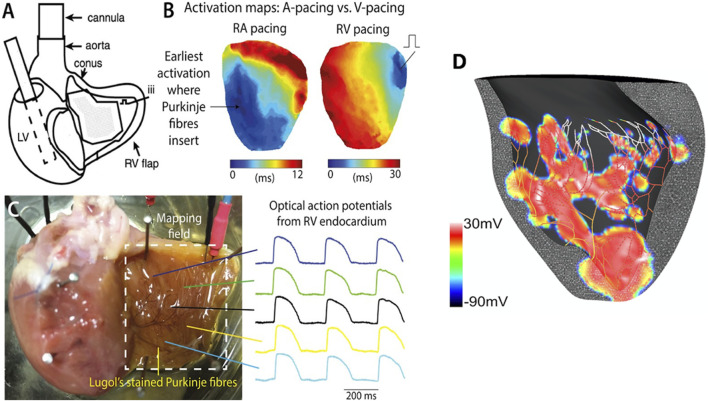
Experimental setup for the global ischaemia-reperfusion optical mapping. **(A)** Schematic diagram of aortic-perfused RV endocardial mapping preparation (modified from Cates et al., AJP Heart 2001). **(B)** Representative RV endocardial activation maps for RA and RV pacing. **(C)** Representative optical APs recorded at multiple sites on RV flap. potential map shows areas of early activation (surrogate of active PMJs) in red (+30 mV). **(D)** Computational model of RV endocardium and Purkinje fibers after His stimulation. This transmembrane voltage map shows areas of early activation (surrogate of active PMJs) in red (+30 mV).

The aortic-perfused right ventricular endocardial mapping preparation was set up as shown in Figure 2A. Our endocardial mapping model was based on the work by Cates et al. (2001), to allow for mapping of the endocardial surface of the RV (Figure 2B). Unlike Cates et al., we did not remove the atria. Cuts were made along the right coronary artery and posterior descending artery, preserving them and maintaining perfusion of the RV, but also allowing the RV to be opened as a flap to allow for endocardial mapping (Figure 2C).

After a period of stabilization, hearts were perfused with a potentiometric dye (di-4-ANEPPS 20–40 µL of 1.25 mg/mL solution; Invitrogen, Carlsbad, CA) and an excitation-contraction uncoupler (10 µM Blebbistatin). Hearts were paced via the high right atrium using a bipolar silver electrode, to allow for physiological activation of the ventricles through the Purkinje system.

Optical mapping of transmembrane voltage was performed as previously described (Ng et al., 2013; Ng et al., 2014; Ng et al., 2016).

Excitation was provided by a 530 nm LED light and emitted light passed through a 650 nm emission filter. A 100 × 100 pixel MiCAM Ultima-L CMOS camera (SciMedia, United States Ltd., CA) was used to detect the optical signals (1,000 frames/sec). Optical signals were recorded during sinus rhythm and right atrial pacing at a range of pacing cycle lengths at baseline (400 ms).

Two groups of hearts were studied experimentally. Seven hearts were subjected to 40 min global ischaemia (no flow ischaemia model) and subsequent reperfusion for 30 min, with recordings at regular intervals during atrial pacing. At fixed intervals, ventricular conduction properties were studied during right ventricular pacing (Figure 2B). Atrial pacing was performed every 10 min, to look at RV activation. For RV pacing to assess CV, this was done at baseline (0 min), peak ischaemia (40 min) and after 30 min reperfusion. A further five hearts were subjected to pretreatment with 50 nM rotigaptide before being subjected to the acute ischaemia-reperfusion protocol. Rotigaptide is a peptide analogue that has been shown to increase gap junction conductance in cardiac muscle cells, acting primarily via connexin 43 (Ng et al., 2016). Since ischaemia shortens action potential duration (APD) in a time dependent manner, APD measurements were taken to confirm that progressive ischaemia had occurred during the experiments (Ng et al., 2014).

### 2.2 Data analysis

Optical mapping data were processed and analyzed as previously described (Laughner et al., 2012). Results were analysed in GraphPad Prism six software (GraphPad Software) and represented as mean ± SEM with p < 0.05 indicating significance. Paired Student’s t-tests were used for relative changes between two groups. * = p < 0.05,** = p < 0.01,*** = p < 0.001.

### 2.3 Computational cable experiments

To isolate effects of ischaemia on conduction velocity, we performed *in silico* experiments in a cable. Since ischaemia is well known to increase extracellular K+, we performed a series of cable experiments with a range of K+ concentrations and determined the resting membrane potential and conduction velocities.

### 2.4 Computational ventricular and purkinje system modelling

Computer simulations were performed on our well established 3D finite element rabbit ventricular mesh incorporating a detailed representation of the Purkinje system, using rabbit ionic models for the ventricles ​(Mahajan et al., 2008) and Purkinje cells ​(Aslanidi et al., 2010). The ventricular mesh comprised about 550k tetrahedral nodes with an average edge spacing of 280 μm. Monodomain simulations were performed with a time step of 25 microseconds. The His-Purkinje mesh was a separate mesh of cubic Hermite one-dimensional elements of 300 μm mean length. PMJs were located at the free ends of the cables, where all nodes in a small volume of myocardium (500 μm radius) were connected to the terminal node of the Purkinje branch through a fixed electrical resistance (*R*_*PMJ*_) (Boyle et al., 2010; Behradfar et al., 2014).

Propagation across the PMJ was set by setting R_PMJ_. Values were chosen so that propagation was asymmetric across the PMJ with retrograde transmission easier with less of a delay (∼1–3 ms) than anterograde transmission (5–8 ms delay)​(Tan et al., 1989) There were about 1,000 PMJs, but the number of functional PMJs (NPMJ) was varied by changing the global value of R_PMJ_ to arrive at eight different percentages of functional PMJs: 5, 10, 15, 20, 25, 50, 80% and 100%. Functional PMJs were distributed almost evenly on the Purkinje branches. Computations were performed using carpentry (Numericor GmbH), which required 1 minute to simulate 30 ms using 12 cores on a 2.2 GHz AMD Ryzen nine desktop computer.

Sinus activation in the ventricles was performed by current stimulation of the proximal His-bundle. CV in the His/Purkinje system was 1.8 m/s while myocardial tissue conductivities were adjusted to reproduce measured healthy CVs, approximately 60–70 *cm/*sec longitudinally and 25–34 *cm/*sec transversely ​(Kelly et al., 2013), while reproducing measured activation times. For His pacing, epicardial activation took about 21 ms, which is in the range measured experimentally (16 ms by electrodes array *versus* 25 ms by optical mapping) (Azarov et al., 2007; Bordas et al., 2011).

Myocardial uncoupling during ischaemia was effected by 25% and 50% reductions in all tissue conductivities. Since neither the time course nor the exact extent of uncoupling are known, we sought to cover a plausible range. Ischaemic cellular electrophysiological changes were as suggested by Tice et al. (2007): maximum conductances of Na and L-type Ca channels reduced by 25%, [K^+^]_e_ increased to 11 mM, and the fraction of ATP-sensitive K^+^ channels increased to 0.045%. The His/Purkinje system was assumed unaffected by ischaemia ​(Argentieri et al., 1990).

### 2.5 Measuring velocity

#### 2.5.1 Experimental activation

Activation patterns of the endocardial surface of the RV free wall were analyzed as represented in Figure 2D. Activation time (AT) was defined as the instant the maximum rate of rise of the upstroke for experimental data. ATs were spatially smoothed and the divergence of the propagation velocity was used to quantify alterations in activation pattern complexity (Fitzgerald et al., 2005).

The approximate area of endocardial surface mapped was 2 × 2 cm. To assess the contribution of changes in propagation across PMJs and the RV activation pattern during ischaemia and reperfusion, the proportion of pixels activated within the first 5 ms was quantified during RA pacing.

For each AT map, the average AT value was subtracted and outliers (more than 2-standard deviations from the mean) were excluded. ATs were averaged over several cycles and finally, spatially filtered by a 2D Gaussian smoothing kernel with a standard deviation of five pixels. For calculating the gradient of AT, a 3 × 3 pixel moving window was applied to the optical image. The gradient was found by fitting a plane to activation time values over that window, using principal component analysis. The gradient was calculated only for windows with less than half of the pixels being outliers.

#### 2.5.2 Computational activation

For computer simulation data, AT for each node on the RV endocardial surface was defined as the time of positive crossing of the −20 mV threshold. For His pacing, the portion of pixels in the first 8 ms was counted, not first 5 ms as with experiment, since <4% of the tissue was ever activated within the first 3 ms, and the higher resolution of the model also influenced this. In our finite element model, the endocardial surface was defined by an unstructured triangular mesh, and the gradient was found by using the gradient of the shape functions.

### 2.6 Velocity and complexity

Velocity at point n was obtained from:

$$v\left(n\right)=\frac{\nabla AT}{{\left|\nabla AT\right|}^{2}}$$

The divergence of the propagation velocity was used to quantify alterations in activation pattern complexity. The divergence of velocity, $\nabla \cdot v\left(n\right)=\frac{{\partial v}_{x}}{\partial x}+\frac{\partial v_{y}}{\partial y}$ was calculated by finding the gradients of each of the components of the velocity field and adding them. The integral of the absolute value of the divergence (IVD) over the endocardial surface was used to quantify overall complexity:

$$IVD=\int_{RV_{endo}}\left|\nabla \cdot v\right|d\Omega$$

## 3 Results

### 3.1 Effect of acute ischaemia–reperfusion on endocardial activation

**FIGURE 3:**
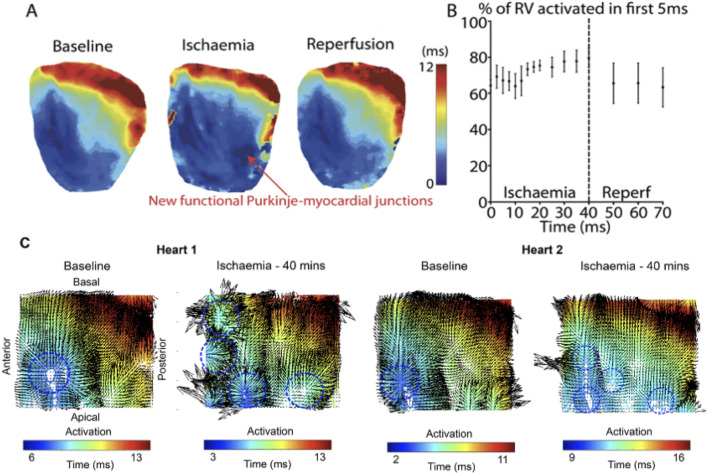
Representative activation maps during ischaemia and reperfusion **(A)** Activation maps following RA pacing during baseline, ischaemia and reperfusion. **(B)** Percentage of RV activated in first 5 ms during ischaemia and reperfusion. Data are presented from four control hearts where there were more frequent recordings during early ischaemia (every 2.5 min during the first 20 min), to provide more detail on the time course of these changes. **(C)** Representative activation maps with local conduction vectors at baseline and after 40 mins ischaemia. At baseline, the earliest activation is at the anterior-apical region of the RV, but multiple areas of early activation can be seen at peak ischaemia. Blue circles indicate breakthorough sites.

Activation maps during baseline, acute ischaemia and reperfusion are shown in Figure 3A. At baseline, a single anterior region of RV endocardium was activated first. With acute ischaemia, the percentage of RV area activated early (i.e., within the first 5 ms) decreased from 64% ± 3% at baseline to 52% ± 8% during early ischaemia (p = 0.04), consistent with slowing of conduction. However, during late ischaemia, extra regions of early activation (e.g., in the posterior RV), consistent with areas of functioning PMJs previously-quiescent under baseline, pre-ischaemic conditions were seen (Figure 3A). The percentage of RV area activated early (0–5 ms) in late-ischaemia (>15 min) was similar to that at baseline, with a maximum of 80% ± 5% RV early activation after 40 min (p = NS vs. baseline; (Figure 3B). The values returned towards baseline following reperfusion, decreasing back to 63% ± 11% of RV early activation after 30 min of reperfusion. Representative activation maps with the divergence maps at baseline and after 40 min are shown in Figure 3C. More early breakthrough sites (indictaed by radiating arrows), consistent with newly activated PMJs, were seen at 40 min of ischemmia than at baseline.

### 3.2 Effects of acute ischaemia-reperfusion in rotigaptide treated hearts

**FIGURE 4:**
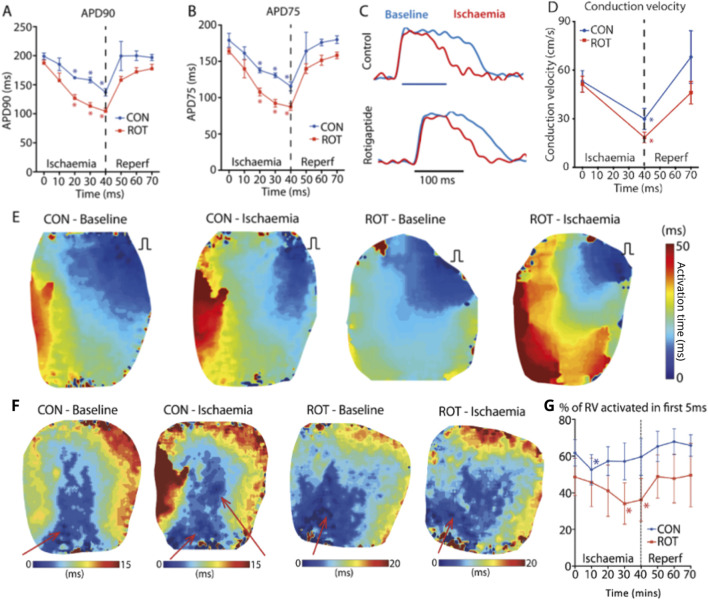
Effect of Gap Junction Enhancer Rotigaptide. Compared to control, rotigaptide shortened **(A)** APD90 and **(B)** APD75 over the course of the ischaemia. **(C)** Representative action potentials at baseline (blue) and 40 min ischaemia (red) for control and rotigaptide treated heart. **(D)** Conduction velocity for control and rotigaptide treated hearts. **(E)** RV pacing activation maps at baseline and 40 min of ischaemia for control and rotigaptide treated hearts. **(F)** RA pacing activation maps at baseline and 40 min of ischaemia for control and rotigaptide treated hearts. There are new areas of early activation (functional PMJs indictaed by red arrow) in a control heart during RA pacing, which was not seen in the rotigaptide treated heart. **(G)** Effect of ischaemia and reperfusion on control and rotigaptide treated hearts on percentage of right ventricle activated within the first 5 ms. Likely because of more functional PMJs during ischaemia, the percentage of RV area activated early (within 5 ms) was maintained around 60% in control group, but not in the rotigaptide group. CON–control; ROT–rotigaptide. Data are for control (n = 7) and rotigaptide (n = 5) groups in their entirety. Asteriskes indicate statistical significance.

We next assessed the effect of gap junction enhancement, with rotigaptide, on RV activation times and patterns during acute ischaemia and reperfusion. During acute global ischaemia, shortening of APD and slowing of conduction were observed, with normalization following reperfusion (Figure 4A-C) with and without rotigaptide. Control APD90 decreased from 199 ± 6 ms to 137 ± 6 ms after 40 min of ischaemia (p < 0.05). This then normalized after 10 min of reperfusion (200 ± 25 ms). Similar trends were also noted in the rotigaptide group. APD90 decreased from 187 ± 5 ms to 104 ± 5 ms after 40 min of ischaemia (p < 0.05) and normalized after 20 min of reperfusion (172 ± 7 ms).

The control CV (Figure 4D) decreased from 53.1 ± 6.5 cm/s to 29.9 ± 6.5 cm/s after 40 min of ischaemia (p < 0.05), which then normalized to 68.1 ± 16.2 cm/s after 30 min of reperfusion. Local CV slowed in the rotigaptide group following 40 min of global ischaemia (baseline 51.3 ± 5.0 vs. ischaemia 18.6 ± 3.3 cm/s, p < 0.05). Figure 4E shows examples of RV activation under RV pacing from which CV measurements were made. With rotigaptide after 40 min of ischaemia, the rotigaptide-treated heart counter-intuitively took longer to activate.

With RA pacing (Figure 4F), the activation of the RV was much faster compared to RV pacing, less than one half of the time. At baseline, rotigaptide treated hearts behaved similarly to control hearts, with the anterior region of the RV endocardium activating first, and the percentage of RV area activated within the first 5 ms decreased during ischaemia. However, unlike the control hearts, no extra regions of early activation were observed, and the paradoxical increase in the percentage of RV area activated within 5 ms in late ischaemia seen in the control hearts was not observed in rotigaptide-treated hearts, with a progressive reduction in percentage of early-activated regions in the RV (baseline: 49% ± 10%; 10 min ischaemia: 46% ± 13%, 20 min ischaemia: 41% ± 14%). The relative decrease in early RV activation appeared to be due to a reduction in the number of functional PMJs, for the same stage of acute ischaemia, when compared to control hearts (Figure 4F). The percentage of early-activated regions in the RV in the rotigaptide group was significantly reduced compared with baseline at 30 and 40 min of ischaemia (both p < 0.05, Figure 4G).

### 3.3 Modelling experiments

#### 3.3.1 Cable propagation

With nominal [K^+^] of 5.4 mM, CV was 51.3 cm/s, the resting membrane potential was −87.15 mV, and the threshold for excitation was 27.1 uA/cm^2^. Increasing [K^+^] to 8 mM yielded a CV of 56.3 cm/s, a resting membrane potential of −76.9 mV, and a threshold of excitation of 19 uA/cm^2^. A further increase of [K^+^] to 12 mM resulted in a slower CV of 41.7 cm/s, a resting level of −66.5 mV, and a excitation threshold of 14.30 uA/cm^2^. With [K^+^] of 8 mM, implementing 25% reductions in Na and L-type Ca conductances reduced CV to 50.8 cm/s, and reducing myocardial coupling by 25% further reduced CV to 44.1 cm/s. Under normoxic conditions, tranverse CV was 24 cm/s, and implementing ischaemic ionic and coupling changes resulted in a CV of 20.5 cm/s.

#### 3.3.2 RV simulation

Anterograde conduction occurred after only a 6% reduction in conductivity.

**FIGURE 5:**
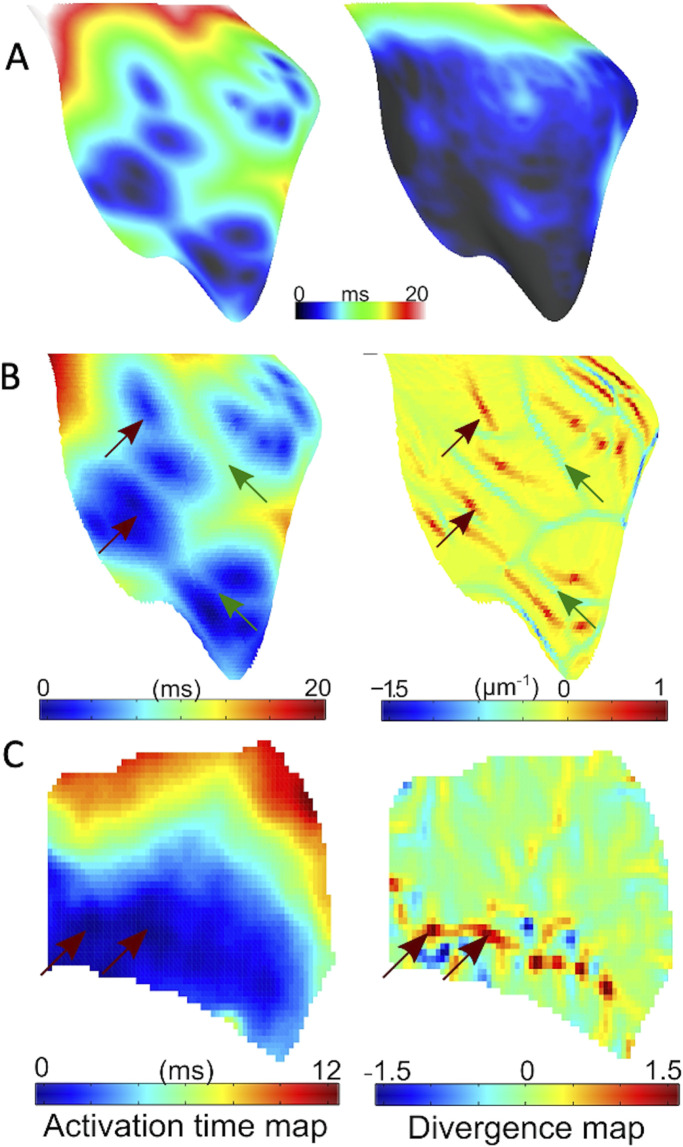
Endocardial activation and divergence maps **(A)** Simulated RV endocardial area isochoronal activation maps during His bundle pacing for 5% (left panel) and 80% (right panel) active junctions. **(B)** Representative simulated activation (left panel) with 10% active junctions, and corresponding endocardial divergence map (right panel). Sites of breakthrough are indicated in red arrows on the activation map. Corresponding points on divergence map have large positive divergence, while green arrows represent wave collisions with matching points indicating a large negative divergence. **(C)** Representative experimental activation (left panel) and corresponding endocardial divergence map (right panel). Arrows point to breakthrough sites on activation maps that match the location of large positive divergence on the divergence map.

Figure 5A depicts the isochronal activation maps on the RV endocardium for low (5%) and high numbers (80%) of active PMJs. More active PMJs account for more breakthrough sites in the model, a reduced endocardial activation time and increased complexity of activation. We simulated ischaemia in the ventricular model and measured local CV following RV endocardial stimulation to emulate experiment. Using an ischaemic ventricular ionic model, the myocardial conductivity and number of active PMJs were varied to evaluate their effects on local apparent RV endocardial CV, that is, the CV obtained from naively differencing local activation times without regard for multiple activation sites. Results of a model without the Purkinje system (zero PMJ) were added to compare the CV derived only from myocardial propagation. Significant increases in apparent local CV because of a greater number of active PMJs implies a contribution of the Purkinje system through retrograde and anterograde conduction, which can be greater than effects of increased conductivity on local CV.

RV endocardial activation duration altered in the range of 13–21 ms for different numbers of active PMJs, while epicardial activation was hardly affected. Figure 5B illustrates the value of divergence on the RV endocardial surface for the model with 5% active PMJs. Figure 5C depicts a sample of the activation map and its associated velocity divergence. In both optical and modelling maps, values of divergence were very small (close to zero) in most regions of the surface where the wave-front propagated normally without collisions. Positive divergence is indicative of breakthroughs and negative divergence identifies wave front collisions.

#### 3.3.3 Activation complexity

**FIGURE 6:**
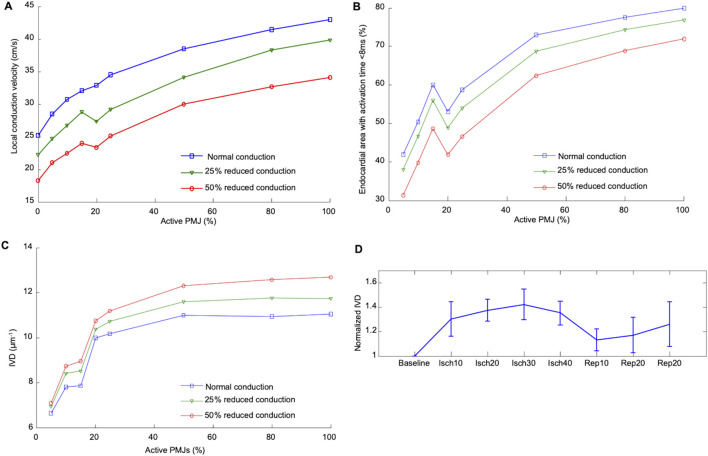
Effect of percentage active PMJs on activation time and conduction velocities **(A)** Alteration in median local CV in ischaemic computer model by conductivity and number of active PMJs following RV pacing. **(B)** Variations in the activated area with changes of percentage of active PMJs and myocardial conductance in computer model of sinus activation. The endocardial area that activated within the first 8 ms mainly was increased by increasing PMJ density and reduced by decreasing myocardial conductivity. **(C)** Calculated IVD relative to number of active PMJs and myocardial conductance in computer model during sinus activation. **(D)** Experimental effect of ischaemia and reperfusion on calculated IVD during atrial pacing.

Results shown in Figure 6 suggest important effects of PMJ density on activation acceleration which was more significant than slowing as result of reduced conductivity. The endocardial area that activated within the first 8 ms mainly increased with greater PMJ density and reduced with decreasing myocardial conductivity. The integral of divergence over the RV endocardial surface was then calculated to quantify differences in propagation patterns. The absolute value of the sum of the divergence over the surface, which represents the complexity of activation pattern, was calculated for optical maps as represented in Figure 6C. Graphs show mean values of IVD, which were normalized to the value at baseline for each heart. Ischaemia increased the IVD, indicative of more breakthrough during ischaemia relative to baseline and reperfusion (Figure 6, n = 4 rabbit hearts). IVD values for different PMJ densities and conductivities are represented in Figure 6C. As the number of active PMJs increased, more breakthroughs and wave collisions occurred and as a result, IVD increased (6.6 at 5% functional vs 11.0 at 80% functional). Reducing ventricular conductivity (50% decrease) did not impact IVD considerably at low PMJ density (7.1 vs 6.4) but it inversely altered IVD with more active PMJs (12.7 vs 11.0). The recruitment of PMJs by changing R_PMJ_ was not spatially uniform, accounting for the non-monotonic curves in Figures 6A–C.

## 4 Discussion

The main findings of this study are: 1) many PMJs are quiescent or non-functioning at rest; 2) acute ischaemia leads to an increase in the number of active PMJs, which consequently resulted in faster total ventricular endocardial activation; and 3) this finding was mitigated by enhancing gap junction coupling using rotigaptide.

### 4.1 Acute ischaemia increases the number of active PMJs

Preclinical data have indicated that many PMJs are non-functioning at baseline and this is thought to be due to source-sink mismatch at these junctions (Mendez et al., 1970; Joyner, 1982; Overholt et al., 1984). This hypothesis is supported by a study in connexin43 knockout mice, where multiple extra epicardial wavefronts were observed when compared to control hearts. It was thought that alterations in source-sink relationships due to Cx43 knockout led to paradoxical conduction across normally quiescent PMJs and resulted in the aberrant activation profiles and wavefront collisions (Morley et al., 2005). Our results suggest that most anterograde PMJ transmission failures occur because of too much coupling, instead of too little. This might be expected since most modulating factors tend to reduce gap junction conductance (increased [Ca_i_^2+^] or decreased pH), and reducing weak coupling would little affect the quiescence of the gap junctions.

Consistent with the above, we showed that acute ischaemia, which causes gap junction uncoupling and a decrease in CV (Janse et al., 1986), was associated with an increase in activation complexity, resulting from activation breakthroughs, which led to a similar paradoxical acceleration in RV activation. We postulate that ischaemia-induced gap junction closure facilitated more successful anterograde PMJ propagation by reducing electrical load at normally quiescent PMJs all along the conduction system, including towards the base. Therefore, despite reduced local CV, the activation time of the total endocardium was shortened. In contrast to the previously mentioned Cx43 knockout study, we analyzed PMJs located at the endocardial surface rather than the epicardial surface, which notably does not contain PMJs and therefore indirectly assesses PMJ function ​(Morley et al., 2005).

Tranum-Jensen et al. reported an intermediate cell type between Purkinje and myocardial cells consisting of distinct morphological features and postulated protection of anterograde propagation ​(Tranum-Jensen et al., 1991). However, in that paper, there was insufficient functional support for what they describe since the tissue environment was not manipulated nor were any electrical propagation delays across the PMJ reported. In contrast, Tan et al. did explore this and they reported that transmission was modulated and could fail depending on changes in potassium concentration and hypoxia (Joyner, 1982). Therefore, electronic interaction is important at the PMJ, whether it be from Purkinje to transitional cell or transitional cell to muscle.

Unfortunately, due to the limitations of even state-of-the-art optical mapping techniques, we were unable to individually visualize PMJs and it must be stated that areas of early activation may be because of several mechanisms including: 1) Increased PMJ activations; 2) Ectopic foci; 3) A wavefront travelling on an epicardium decoupled from the endocardium except at distinct regions. Our modelling data showed that the activation times were confluent with sinus activation, ordered and consistent, which argues against ectopic foci (point 2). Point 3 cannot explain our results. If ischaemic uncoupling essentially divided the wall into epicardial and endocardial regions with a few discrete connection points resulting in more breakthroughs, the CV would still by reduced and result in longer activation. Arguing that propagation in the thin layer is faster than the complete wall is unfounded, as plane waves will eventually result in both cases. Therefore, we believe that increased PMJ activation remains the most plausible option.

### 4.2 Gap junction enhancement prevents PMJ activation during ischaemia

Simulations in the cable indicated that ischaemia could lead to increased early activation sites via K^+^ concentration if it was the dominant change, by reducing current thresholds for excitation. However, other ischaemic effects will cause an overall decrease in CV. Increased tissue excitability could also explain the findings of increased early activation sites: it may be possible that the reduced current thresholds for excitation, as shown in the cable simulations, and the improved source-sink match at the PMJs due to change in gap junction coupling are both important in explaining the observed changes in RV activation pattern during ischaemia. In the organ simulations, however, reduced current thresholds were not necessary. The Purkinje system is less affected by schema than myocardial tissue (Fenoglio et al., 1976), with CV remaining unaffected even 1 day after ligation in a dog model (Argentieri et al., 1990).

We further explored our hypothesis by assessing the effect of pharmacological modification of gap junction coupling using rotigaptide, which enhanced gap junction coupling during ischaemia. In hearts treated with rotigaptide, we postulate that PMJs which were usually quiescent, but became functional during ischaemia, were again rendered quiescent since the number of breakthroughs decreased. These findings support our hypothesis changes in gap junction coupling can alter PMJ anterograde impulse propagation. Under normal conditions, there is a high level of safety (redundancy) in the number of open gap junctions; thus, rotigaptide did not affect tissue conduction velocity. Treatment with rotigaptide during ischaemia reduced the number of new functional PMJs, mitigating ischaemia-induced gap junction uncoupling, and maintained the level of source-sink mismatch present during baseline conditions.

The divergence maps could also have been used to build estimates of the Purkinje network as has been performed previously (Barber et al., 2021). Breakthrough sites are clearly identified, as in Figure 5, and show activations in the lower region which anatomically correspond to the region around the moderator band, where PMJs are know to be located. The greater IVF during ischaemia results from more breakthroughs, and allows a more complete depiction of the Purkinje network.

### 4.3 Local increases in CV by gap junction enhancement were not seen endocardially

Rotigaptide has previously been shown to improve local CV during acute ischaemia and in the context of metabolic stress by maintaining gap junction coupling (Eloff et al., 2003; Ng et al., 2016). However, this effect was not observed in our study. It should be noted that we used a novel endocardial mapping preparation, whilst the other studies on the effects of rotigaptide were based on epicardial mapping. Local apparent endocardial CV was calculated based on a 2D projection of 3D conduction and, therefore, indicative of overall endocardial activation velocity. In rabbit hearts, Purkinje fibers only penetrate a shallow subendocardial layer, and therefore this measure partially depends on activation through the Purkinje system. This may explain why rotigaptide did not result in an improvement in CV that has been reported by other groups who analyzed the epicardial surface, which does not contain PMJs, and where the calculated epicardial CV is predominantly based on myocardial wavefront propagation ​(Dhein et al., 2010).

### 4.4 Modelling insight

To ascertain whether our observed findings were due to enhanced PMJ function, we performed a series of modelling experiments mimicking the experimental set up. Myocardial conductivity and the number of active PMJs were varied in the model to evaluate effects on local apparent RV endocardial CV. A model without the Purkinje system (zero PMJs) allowed comparison of the CV derived solely from endocardial activation times. Significant increases in local apparent CV because of more active PMJs implied a contribution of the Purkinje system through both retrograde and anterograde conduction, which could be greater than effects of conductivity on local CV. Moreover, the changes in apparent endocardial CV that occurred with different PMJ densities, implied that measured CV is not solely dependent on myocardial cell-to-cell conduction but also via the Purkinje system as well. Comparison of modeling and experiment results in Figures 5, 6 suggests a potential mechanism for accelerated activation during acute ischaemia. Increasing the number of active PMJs in the model accelerates endocardial activation and compensates for the slowing of propagation. Thus, the area activated in a model with reduced conductivity and a high percentage of active PMJs (ischaemia) can be equal to or larger than the area activated under normal conditions. Note that in Figure 6, the curve is not monotonic due to the nonuniform distribution of active PMJs and a random sampling of them. At an active PMJ level of 15%, the active PMJs were very well separated, resulting in a large area activated through local CV conduction.

Normally, severe uncoupling conditions cause discontinuity and meandering of activation wavefronts, where islands of uncoupled cells enforce twisting of the activation wavefront. However, we do not believe that this is the reason underlying the observed increased activation complexity during ischaemia since the CV reduced to 20–30 cm/s due to both reduced excitability and reduced coupling, which is a modest reduction compared to the CV measured during critical gap junction uncoupling. In addition, CV completely recovered following reperfusion in our biological experiments. Therefore, we conclude that the observed increase in activation complexity under ischaemia can be attributed to function of normally inactive PMJs. In addition, ischaemic hearts treated with rotigaptide could be analogous to the model of increased conductivity and few active PMJs, which could lead to lower local endocardial CVs.

### 4.5 Clinical implications

The Purkinje system is pivotal in the pathogenesis of several pathological ventricular arrhythmias including Purkinje-triggered VF, bundle branch reentry and fascicular VT (Haissaguerre et al., 2016). Since we were able reduce the number of active PMJs during acute ischaemia pharmacologically by enhancing gap junction function, this could be a viable therapeutic strategy to reduce the propagation of Purkinje system arrhythmias into the ventricular myocardium, either as a temporizing measure prior to definitive management (catheter ablation) or even periprocedurally as an adjunctive therapy.

### 4.6 Study limitations

Our interpretation of data is based on relative changes in activation, which was used as a surrogate to indicate active PMJ density. Nevertheless, with current state-of-the-art optical mapping techniques, it is impossible measure individual PMJs and, as such, indirect evidence of activation is taken from clusters or groups of PMJs becoming active and manifesting as a breakthrough. However, this method does have significant advantages, in that it allows for simultaneous mapping of the entire RV endocardial surface to look at changes RV activation patterns and infer propagation across multiple PMJs simultaneously. Secondly, our model of the PMJ replicates transmission behavior at junctions, but we were not able to study complete effects of ischaemia using this simple representation. For example, the models used for this research did not account for myocardial heterogeneity, which contributes to disparate response of different layers of ventricular wall to ischaemia. Thirdly, we did not include any ischaemic effects on the Purkinje system since it is known to be more resistant to ischaemia.

In this study, our objective was to investigate the changes in acute ischaemia only and the reversibility of these changes following reperfusion, and we were therefore not able to study the changes associated with longer durations of ischaemia. In this experiental model, we did not bubble the physiological solution with nitrogen. There was likely a reduced depth of ischaemia than if we had bubbled with nitrogen, though the slowing of conduction and changes in APD confirm that there was significant myocardial effects.

## 5 Conclusion

In this study, using an endocardial mapping preparation that allowed for the interrogation of propagation patterns across PMJs, we found behaviour consistent with a significant majority of PMJs at baseline being quiescent. Activation of these normally-quiescent PMJs by gap junction uncoupling during acute ischaemia could explain a paradoxical acceleration in RV endocardial activation, and an increased activation complexity. Pharmacological gap junction modulation significantly altered propagation patterns during ischaemia, presumably through changing source-sink mismatch, and could be a therapeutic strategy for arrhythmia control.

## Data Availability

The raw data supporting the conclusions of this article will be made available by the authors, without undue reservation.
